# Association Between Portal Vein Color Doppler Findings and the Severity of Disease in Cirrhotic Patients With Portal Hypertension

**DOI:** 10.5812/iranjradiol.4489

**Published:** 2011-12-25

**Authors:** Puneet Mittal, Ranjana Gupta, Gaurav Mittal, Vishal Kalia

**Affiliations:** 1 Department of Radiodiagnosis, Punjab Institute of Medical Sciences, Jalandhar, India; 2 Department of Medicine, Punjab Institute of Medical Sciences, Jalandhar, India; 3 Department of Radiodiagnosis, Dayanad Medical College and Hospital, Ludhiana, India

**Keywords:** Child, Liver Cirrhosis, Hypertension, Portal

## Abstract

**Background:**

Doppler ultrasound is the accepted gold standard for assessing direction of flow in the portal vein (PV). Moreover, it is non-invasive; therefore, it is well accepted by the patients and does not interfere with flow hemodynamics.

**Objectives:**

The present study was aimed to evaluate the association between color Doppler findings and the severity of portal hypertension in patients with cirrhosis.

**Patients and Methods:**

The study group included 50 patients referred for ultrasound (US) evaluation over a period of six months from March to August, 2007. The patients were divided into three groups (Child’ A, B and C) based on Child Pugh classification. The direction of flow in the main portal vein (hepatopetal or nonhepatopetal) and peak venous velocity (PVV) in the main portal vein were measured and correlated with the presence or absence of ascites, splenomegaly, splenic and esophageal varices (assessed by Doppler US). These findings were correlated with clinical features and laboratory findings (using Child Pugh’s criteria).

**Results:**

There was significant association between the decrease of peak portal venous velocity (PVV) and the increase in Child Pugh score. Hepatofugal flow was seen only in patients with more advanced disease. There was also significant association between PVV and splenic varices and ascites, while PVV was not affected by the presence or absence of esophageal varices or splenomegaly. Presence of a recanalized umbilical vein (UV) was associated with increased PVV even in advanced disease.

**Conclusions:**

Color Doppler is an excellent modality for detecting and characterizing the complex hemodynamics of portal hypertension in cirrhosis and they correlate with the clinical stage of disease.

## 1. Background

Cirrhosis, as defined by the World Health Organization (WHO), is “a diffuse process characterized by fibrosis and conversion of normal liver architecture into structurally abnormal nodules” [1]. Normal portal vein pressure is 5-10 mmHg. Portal hypertension is defined as “a wedged hepatic vein pressure or direct portal vein pressure of more than 5 mmHg greater than the inferior vena cava pressure or surgically measured portal venous pressure of greater than 30 cm water” [2]. Cirrhosis is by far the most frequent cause of portal hypertension. Increased vascular resistance is usually present at multiple anatomical sites simultaneously [3]. The most widely used tool for predicting the prognosis in cirrhosis is the Child Pugh system. Child and Turcotte first introduced their scoring system in 1964 which was subsequently revised by Pugh in 1973 [4].

Doppler ultrasound (US) has been accepted as the gold standard in assessing the direction of flow in the portal venous system. Moreover, being non-invasive, it does not interfere with flow hemodynamics during measurements.

## 2. Objectives

The aim of the present study was to evaluate the association between direction of blood flow in the main portal vein and the maximum portal vein velocity with the presence or absence of ascites, splenomegaly, splenic and esophageal varices and to evaluate the association between Doppler findings and clinical features and laboratory investigations using Child Pugh’s criteria.

## 3. Patients and Methods

This study was carried out over a period of 6 months on 50 patients with the clinical diagnosis of cirrhosis with portal hypertension. A composite assessment of the patient’s history, findings on physical examination, laboratory investigations and results obtained from Doppler US examination was made. All parameters were obtained by one operator. The study was approved by the university and written informed consent was obtained from the patients. Patients with grade 3 and 4 encephalopathy were excluded because of inability to fully cooperate in the examination. The diagnosis of cirrhosis with portal hypertension was based on a combination of clinical data such as jaundice, ascites, muscle wasting, cutaneous spider angiomas, ecchymosis, palmar erythema and flapping tremors; laboratory data such as decreased serum albumin and prolonged prothrombin time; and US findings such as coarsened echo texture and irregular liver surface. Endoscopy was not performed in all patients. The patients were divided into three groups; namely, Child’s A, Child’s B and Child’s C cirrhosis groups using Child-Pugh criteria which was based on the assessment of a combination of laboratory parameters (serum bilirubin, serum albumin and prothrombin time international normalized ratio [INR]) and clinical parameters (encephalopathy [5] and ascites) (Table 1) (Box).

**Table 1:** Child Pugh’s Scoring System

	**1 Points**	**2 Points**	**3 Points**
Encephalopathy	None	Grade 1-2	Grade 3-4
Ascites	Absent	Slight	Moderate
Total bilirubin, (mg/dL)	< 2.0	2.0-3.0	> 3.0
Serum albumin, (g/dL)	> 3.5	2.8 - 3.5	< 2.8
Prothrombin time, s	< 4	4-6	> 6
prothromibin time, INR a, s	< 1.7	1.7-2.3	> 2.3

^a^ Abbreviaton: INR, international normalized ratio

**Box:** Child Pugh Grades of Encephalopathy and Class Scoring

**Grades of Encephalopathy**
0: Lack of detectable changes in personality or behavior. No asterixis is detected.
1: Trivial lack of awareness, shortened attention span, sleep disturbance and altered mood. Asterixis may be present.
2: Lethargy, disorientation to time, amnesia of recent events, impaired simple computations, inappropriate behavior, slurred speech. Asterixis is present.
3: Somnolence, confusion, disorientation to place, bizarre behavior, clonus, nystagmus, positive Babinski sign. Asterixis usually absent.
4: Coma and lack of verbal, eye and oral response
**Child Pugh Scoring Classes**
Class A: 5-6 points
Class B: 7-9 points
Class C: 10-15 points

All the patients were examined with Philips Envisor Whole Body Color Doppler machine with multi-frequency transducer ranging from 2 to 5 MHz. Examination was carried out after a minimum of 8 hours overnight fasting. With the patient in the supine position, assessment of the size and echo pattern of the liver was made by gray scale sonography using a combination of subcostal and intercostal approaches. Liver span was measured in the midclavicular line. The normal liver’s echo pattern is homogeneous. A diffuse coarse echo pattern with surface irregularity and nodularity was considered abnormal. Collaterals were assessed using color Doppler at the splenic hilum, at the gastroesophageal junction (esophageal varices), in the ligamentum teres (for recanalized umbilical vein [UV]) and in the gallbladder bed. Ascites was considered minimal if it was limited to the pelvis or hepatorenal recess. Further than that was described as abundant. Spleen size was measured by measuring its longest longitudinal dimension. A spleen span of more than 12 cm was considered enlarged.

Using intercostal approach, color Doppler was used to evaluate the direction of the flow in the main portal vein i.e. hepatopetal/nonhepatopetal (hepatofugal or bidirectional) and the peak venous velocity (PVV) with subjects in the supine position during suspended inspiration. For measurement of the velocity, the angle of insonation was always less than 60 degree. The reason for selecting the intercostal approach was to obtain the correct angle of insonation. For an adequate Doppler signal, the direction of flow should be as parallel to the direction of US beam as possible. This may only be achieved using intercostal approach. As the portal velocity is often reduced in portal hypertension, it is important to obtain the correct angle of insonation. Unpaired t test and one way ANOVA were used for comparison of the means, P < 0.05 was considered as statistically significant.

## 4. Results

The mean age of the patients in this study was 45 years. Maximum of the patients were in the 31-40 years age group. Sixty six percent of the patients were men with a male to female ratio of 1.94:1. The maximum number of the patients (42%) belonged to Child’s C group (Table 2).

**Table 2:** Gender Distribution of the Subjects in Different Child’s Scores

	**Cases, No.**	**Child’s A, No.**	**Child’s B, No.**	**Child’s C, No.**
Male	33	9	11	13
Female	17	4	5	8
Male/Female	1.94	2.25	2.20	1.63
Total	50	13	16	21

Using one way ANOVA, there was a statistically significant decrease of albumin levels (F = 92.9, P < 0.0001) from Child’s A (3.61 ± 0.19 g/100 mL) through Child’s B (3.21 ± 0.22 g/100 mL) to Child’s C (2.56 ± 0.25 g/100 mL) group. In addition, there was a statistically significant increase in bilirubin levels (F = 44.67, P < 0.0001) and prothrombin index (PTI) (F = 86.44, P < 0.0001) from Child’s A through Child’s B to Child’s C group (Table 3).

**Table 3:** Laboratory Parameters in Child’s A, B and C Groups With Comparative P Values

	**Overall**	**Child’s A**	**Child’s B**	**Child’s C**	***P* value**
Albumin, mg/100 mL, Mean ± SD	3.05 ± 0.50	3.61 ± 0.19	3.21 ± 0.22	2.56 ± 0.25	< 0.0001
Bilirubin, mg/100 mL, Mean ± SD	3.44 ± 2.46	0.81 ± 0.18	2.89 ± 0.31	5.58 ± 2.22	< 0.0001
PTI a, INR a, Mean ± SD	1.6 ± 0.5	1.18 ± 0.12	1.28 ± 0.12	2.1 ± 0.30	< 0.0001

^a^ Abbreviation: INR, international normalized ratio; PTI, prothrombin index

Overall six patients (12%) among a total of fifty had non hepatopetal flow (hepatofugal/bidirectional), four of them (8%) showed continuous hepatofugal flow and two patients (4%) showed bidirectional flow. Hepatofugal or bidirectional flow was seen only in Child’s C group patients. Three patients (6%) had portal vein thrombus and no flow was detected in the main portal vein, while two patients (4%) had portal cavernoma formation (Table 4). Using one way ANOVA, there was a significant fall in the average PVV from Child’s A to Child’s C group patients (F = 29.87, P < 0.0001). So there was a significant fall in PVV with the increasing severity of the grade of cirrhosis (Table 5).

**Table 4:** Pattern of Flow in the Main Portal Vein in Child’s Groups

	**Overall, No.**	**Child’s A, No.**	**Child’s B, No.**	**Child’s C, No.**
Hepatopetal	39	13	14	12
Hepatofugal	4	0	0	4
Bidirectional	2	0	0	2
PV a thrombosis	3	0	1	2
Portal cavernoma	2	0	1	1

^a^ Abbreviation: PV, portal vein

**Table 5:** Comparison of Average Peak Venous Velocity in the Main Portal Vein in Child’s A, B and C Groups

	**Patients, No.**	**Average PVV a, b, cm/s, Mean ± SD**
Total	39	14.72 ± 4.05
Child’s A	13	18.33 ± 2.22
Child’s B	14	14.59 ± 3.57
Child’s C	12	10.96 ± 2.33

^a^ Abbreviation: PVV, portal venous velocity

^b^ P < 0.0001

Patients with hepatofugal flow had significantly higher bilirubin levels, significantly lower albumin levels and also significantly prolonged PTI compared to patients with hepatopetal flow. Encephalopathy and ascites were also more commonly seen in the presence of hepatofugal flow, but that was statistically significant for encephalopathy (Table 6).

**Table 6:** Distribution of Laboratory and Clinical Parameters of Child Pugh Score in Patients With Hepatopetal and Hepatofugal Flow

	**Hepatofugal**	**Hepatopetal**	***P* value**
Albumin, mg/100 mL, Mean ± SD	2.35 ± 0.13	3.21 ± 0.42	0.0002
Bilirubin, mg/100 mL, Mean ± SD	9.18 ± 0.17	2.54 ± 1.48	< 0.0001
PTI a, INR a, Mean ± SD	2.4 ± 0.2	1.5 ± 0.4	< 0.0001
Encephalopathy, No (%)	2 (50)	2 (5.1)	0.0025
Ascites, No (%)	4 (100)	29 (74.4)	0.258
Child’s score, Mean ± SD	12.75 ± 0.83	8.38 ± 2.30	0.0006

^a^ Abbreviation: INR, international normalized ratio; PTI, prothrombin index

As expected, patients with hepatofugal flow had a significantly higher Child’s score in comparison to patients with hepatopetal flow (P < 0.01).

Splenic varices were the most common varices detected by color Doppler (seen in 82%). Overall, 84% had one or more than one collaterals detected on color Doppler (Table 7). The mean PVV (14.05 ± 4.13) was significantly lower in patients with splenic varices compared to those without splenic varices (17.77 ± 1.68) (P < 0.05). Ascites was also associated with a significant reduction of PVV (P < 0.01).

**Table 7:** Distribution of Various Collaterals Detected in Child’s A, B and C Groups b

	**Total**	**Child’s A**	**Child’s B**	**Childs’s C**
Patients, No.	50	13	16	21
Varices, No.	8	3	3	2
Splenic varices, No.	41	10	13	18
Esophageal varices, No.	7	1	3	3
Recanalized UV a, No.	3	0	1	2
Gallbladder bed varices, No.	1	0	1	0
One or more than one varices, No (%)	42 (84)	10 (76.9)	13 (81.25)	19 (85.7)

^a^ Abbreviation: UV, umbilical vein

^b^ No significant difference between Child’s A vs. B and Child’s B vs. C with P > 0.05

The mean PVV was lower in patients with esophageal varices (14.62 ± 2.62) compared to those without esophageal varices (14.74 ± 4.25). However, this difference was not statistically significant (P > 0.05). Recanalized UV was seen only in patients of Child’s B and C groups. In these two groups, patients with recanalized UV had a significantly higher mean PVV as compared to those without.

Using multiple linear regression analysis, we compared six variables (liver span, spleen span, splenic varices, esophageal varices, ascites and PVV) with patients’ Child’s score in 39 patients with hepatopetal flow. Only PVV and ascites showed significant correlation with Child’s score (P < 0.0001).

## 5. Discussion

Cirrhosis represents the final common result of a variety of insults to the liver and it is the most common cause of portal hypertension [3]. Assessment of prognosis is an important factor in medical decision making. The Child’s classification modified by Pugh et al. [4] has been established as a valuable indicator of prognosis. Study of portal hemodynamics is important as it provides further insight into the pathophysiology of the disease and helps explore new therapeutic alternatives.

Measurement of the portal vein pressure by the direct method, though exact, is an invasive procedure and the complications cannot be excluded. Duplex US has been used as a non-invasive method for the evaluation of portal hemodynamics. It is a safe, painless and repeatable method which is well accepted by the patients and is also relatively inexpensive [6].

The disadvantage of duplex US is its operator dependence. For accurate and repeatable measurements, precise localization of the vessel and a proper angle of insonation is important. Other limitations include obesity, excess bowel gas, massive ascites and respiratory movements which preclude a proper Doppler examination.

**Figure 1:**
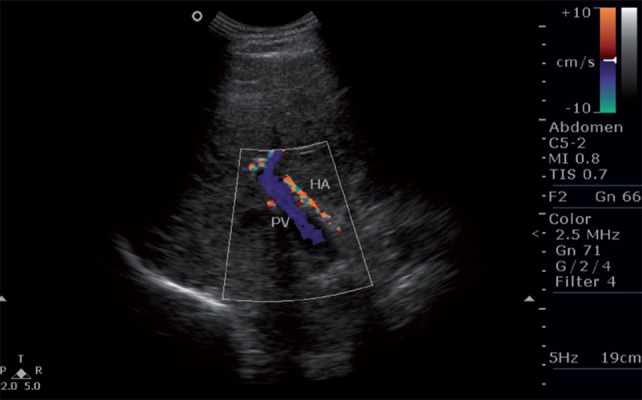
A 55-year-old male patient with cirrhosis. Color Doppler image shows hepatofugal flow in the portal vein. HA, hepatic artery; PV, portal vein

**Figure 2:**
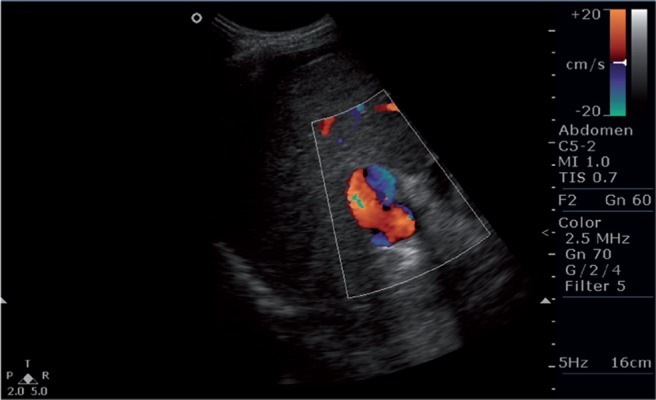
Color Doppler image showing bidirectional flow in the portal vein.

Cirrhosis is associated with increased intrahepatic resistance. This increased resistance manifests as a progressively increasing resistance to the hepatopetal flow in the main portal vein. This increased pressure in the portal vein promotes the opening up of various collateral pathways. These hemodynamic events are responsible for the progressive fall in the portal venous velocity with an increasing severity of the portal hypertension [7]. Furthermore, collaterals help to drain blood away from the portal vein. The reversal of blood flow in the portal system develops when intrahepatic resistance increases over the resistance of portosystemic collaterals. In the present study, all patients with Child’s A cirrhosis had hepatopetal flow and none had hepatofugal flow. In the Child’s B group also, none of the patients had hepatofugal flow. In the Child’s C cirrhosis group, four patients had hepatofugal flow and two patients had bidirectional flow (Figure 1 and Figure 2). So overall six patients (12%) among the total of fifty had non hepatopetal flow and four patients (8%) showed continuous hepatofugal flow and two patients (4%) showed bidirectional flow. Similar patterns have been reported by Von Herbay et al. [8]. However Wachsberg et al. [9] pointed out that the prevalence of hepatofugal flow varied between 3% and 23% in the literature which they attributed the differences to the proportion of patients with advanced disease and to whether the hepatofugal flow was evaluated in the main portal vein only versus its major tributaries as well.

On the whole, the incidence of non hepatopetal flow was 28.5% in Child C cirrhosis group with 19% of the patients showing total hepatofugal flow (Child’s C vs. Child’s A and B, P < 0.05 for hepatofugal flow). Similarly, Gaiani et al. [10] also observed the hepatofugal flow to be more prevalent in patients with Child’s B and C than those with Child’s A cirrhosis (P < 0.02). In the present study, significant higher Child’s score was found in patients with hepatofugal flow (12.75 ± 0.83) compared with patients with hepatopetal flow (8.38 ± 2.30), (P < 0.01) which is similar to Gaiani et al.’s study [9] which reported that Child Pugh score was significantly higher in patients with hepatofugal flow (8.4 ± 2 vs. 7.2 ± 2.2; P < 0.01).

The finding of a reversed flow in the portal venous system is important for understanding the clinical picture of a cirrhotic patient since hepatic encephalopathy may be explained on the basis of large hepatofugal collaterals. Our study shows that the prevalence of reversed flow was significantly high in patients with Child’s C cirrhosis. Similarly, the Child’s score was significantly higher in patients with a reversed flow. This indicates that with reversal of flow, there is more significant impairment of liver function and hence poor Child’s score. It also indicates that with progressive damage to the liver parenchyma, there is consequent increase of intrahepatic resistance. The derangement in ammonia metabolism consequent to liver failure and diversion of a large amount of portal flow into the systemic circulation also results in a significant increase in blood ammonia levels. This correlates with higher trends of hepatic encephalopathy in our patients with a reversed flow. In the present study, among four patients with hepatofugal flow, encephalopathy was present in two patients (50%), while in patients with hepatopetal flow, encephalopathy was seen in only 5.1% of the patients (P < 0.01). Gaiani et al. [10] similarly noted that spontaneous hepatic encephalopathy was significantly more frequent in patients with hepatofugal flow in the portal system (21% vs. 7.2%; P < 0.05).

**Figure 3:**
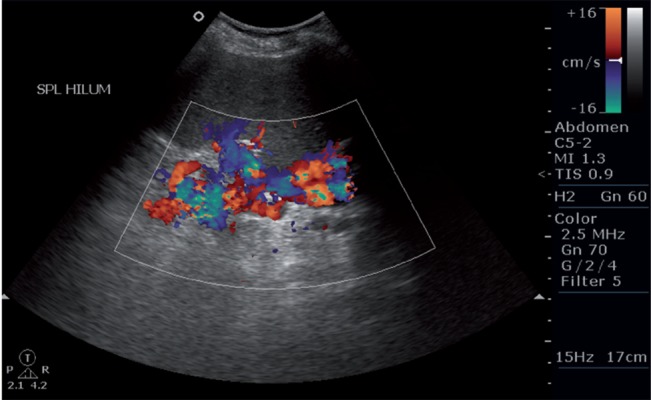
Color Doppler image showing splenic varices.

Cirrhosis is characterized by the presence of extensive fibrosis and numerous regenerative nodules replacing the normal liver parenchyma. So it is logical that an increase in Child’s grade of cirrhosis, the intrahepatic resistance to portal flow increases. This manifests as a decreased peak velocity in the portal vein with the increasing severity of cirrhosis. In the present study, the average of peak velocities in the main portal vein was studied in Child’s A, B and C cirrhosis patients with a hepatopetal flow. Out of 39 patients with hepatopetal flow, the average peak velocity in 13 patients with Child’s A cirrhosis was 18.33 ± 2.22. The average peak velocity in 14 patients with Child’s B cirrhosis was 14.59 ± 3.44, while the average peak velocity in 12 patients with Child’s C cirrhosis was 10.96 ± 2.24. It was observed that the average peak portal velocity in the Child’s B cirrhosis group was significantly lower than that in the Child’s A group (P < 0.01). In addition, the average peak velocity in the Child’s C cirrhosis group was significantly lower than that in the Child’s B cirrhosis group (P < 0.01). Similar trends have been reported in the literature [11][12][13]. The present study could establish the association between the fall in portal velocity and the presence of ascites. In the present study, among the 39 patients with hepatopetal flow, the average peak portal velocity among 31 patients (71.49%) with ascites was 13.60 ± 3.56, while the average peak portal velocity among patients without ascites was 19.15 ± 1.99. This difference is statistically significant (P < 0.01). A similar trend was also reported by Chawla et al. [11] and Shi et al. [13]. This is logical in view of the fact that presence of ascites usually indicates a more advanced Child’s grade of cirrhosis and we have already observed that the increase in Child’s grade of cirrhosis is associated with a statistically significant decrease in the peak portal velocity . In the present study, one or more collateral varices were detected in 42 of 50 (84%) patients. Splenic varices were seen in 82%, esophageal varices in 8%, recanalized UV in 6% and gallbladder bed varices in 2% of the patients. Thus, splenic varices were the most common type of collaterals detected in the present study (Figure 3) and similar observations have been made in other studies. Hepatofugal flow was only seen among patients with splenic varices (4 of 41 patients, 9.76%). On the other hand, none of the patients without splenic varices showed a hepatofugal flow. Among patients with hepatopetal flow, the mean PVV (14.05 ± 4.13) was significantly lower in patients with splenic varices in comparison to those without splenic varices (17.77 ± 1.68) (P < 0.05). Ohnishi et al. [14] found a hepatofugal flow in 50% of patients with splenic varices while none of the patients without splenic varices had a hepatofugal flow. In their study, among patients with hepatopetal flow, portal venous velocity among patients with splenic varices was significantly lower than in those without splenic varices (P < 0.05). Thus, results of the present study are in agreement with Ohnishi et al.’s study [14] mentioning that decreased portal velocity and hepatofugal flow are associated with the presence of splenic varices. This is logical in view of the fact that splenic varices provide an alternate route for drainage of the portal venous flow, thereby diminishing the hepatic flow and thereby the portal velocity. With progression, this may lead to hepatofugal flow.

In the present study, the mean PVV was lower in patients with esophageal varices (14.62 ± 2.62) as compared to those without esophageal varices (14.74 ± 4.25). However this difference was not statistically significant (P > 0.05). Taourel et al. [15] also observed the absence of correlation between the degree of portal hypertension and the development of esophageal varices. The lack of relationship between portal hemodynamic parameters and the development of esophageal varices reflects the fact that, unlike some other collaterals, gastroesophageal collaterals are unable to drain an amount of blood sufficient to significantly lower the portal flow.

**Figure 4:**
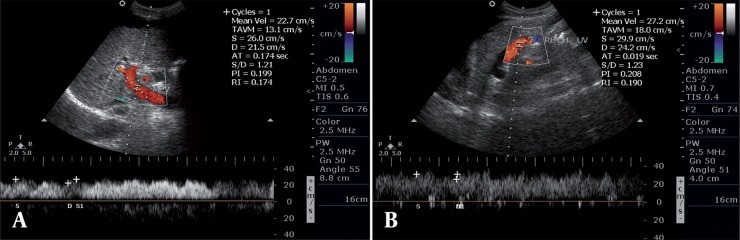
A, Spectral Doppler image showing hepatopetal flow in the portal vein with PVV of 26 cm/s; B, Spectral Doppler image showing continuous hepatofugal flow in recanalized UV.

Using multiple regression analysis, comparison of six variables (liver span, spleen span, splenic varices, esophageal varices, ascites and PVV) showed that only PVV and ascites have significant correlation with Child’s score, thereby highlighting the importance of PVV in predicting the severity of the disease. However, hemodynamics of portal hypertension are quite complex and portal velocity alone does not always reflect the degree of hepatic damage and hepatic encephalopathy. This is reflected by three patients with recanalized UV in our study (two with Child’ C and one with Child’s B cirrhosis). The average PVV in patients with recanalized UV was 17.51 ± 8.33, while the average PVV in Child’s B and C patients without recanalized UV was 12.32 ± 2.14. The difference was statistically significant (P < 0.01). Encephalopathy was present in 33% of cases with recanalized UV while it was seen in 8.5% of cases without recanalized UV (P < 0.05). Others [15][16][17][18] have made similar trends that recanalized UV is associated with increased portal velocity. Similarly Gupta et al. [18] observed that the patients with portosystemic encephalopathy had a significantly greater prevalence of patent UV than those without encephalopathy. Therefore, presence of this collateral is associated with increased flow velocities in the main portal vein (Figure 4). Recanalized UV is also associated with a higher incidence of encephalopathy. These findings are in agreement with previous studies. This is consequent to the fact that a recanalized UV drains blood through the left branch of the portal vein, therefore it does not drain flow away from the main portal vein. Besides, because it is an efficient collateral, it is usually associated with an increased incidence of hepatic encephalopathy and a higher Child’s grade. Therefore, the evaluation of portal velocity in cirrhotic patients may provide misleading results if the paraumbilical vein is patent, underestimating the degree of portal hypertension. So these collaterals should always be searched for, especially in patients with a high portal velocity.

Limitations of the present study include that diagnosis of cirrhosis and portal hypertension was based on the combination of clinical, laboratory and US findings. Liver biopsy or objective measurements were not done to prove the diagnosis. This could lead to excluding patients with early disease and those with atypical findings. In addition, no follow-up was done to evaluate if Doppler parameters correlated with the final outcome or not.

In conclusion, color Doppler is an excellent modality for delineating the complex hemodynamics of portal hypertension in cirrhotic patients. Doppler findings of direct and velocity of flow in the main portal vein correlate with clinical parameters using Child Pugh score. Presence of splenic varices and ascites also correlate with the decrease in portal velocity, while splenomegaly and esophageal varices do not. Presence of recanalized UV may be associated with an increased PVV and is associated with the increased incidence of encephalopathy and a higher Child’s grade.
